# Keratinized mucosa augmentation guided by double xenogeneic collagen matrix membranes around implants in the posterior mandible: A case report

**DOI:** 10.1097/MD.0000000000023609

**Published:** 2021-01-22

**Authors:** Chunyu Han, Qing Cai, Baosheng Li, Yuyang Li, Yanqun Liu, Haina Yu, Mengxiao He, Weiyan Meng

**Affiliations:** School and Hospital of Stomatology, Jilin University, Department of Dental Implantology, Jilin Provincial Key Laboratory of Tooth Development and Bone Remodeling, Changchun, Jilin Province, China.

**Keywords:** keratinized mucosa augmentation, xenogeneic collagen matrix membranes, implant health

## Abstract

**Rationale:**

Traditional free gingival graft (FGG) technique is usually used for patients with insufficient peri-implant keratinized mucosa. However, this technique often requires a second surgical area which increases the pain as well as the risk of infection in patients. Xenogeneic collagen matrix (XCM) membrane technique can obtain good results for keratinized mucosa increment.

**Patient concerns:**

The patient was a 66-year-old healthy female with loss of left mandibular first molar and second molar (FDI #36, #37) for 5 years. Two implants were placed submucosally for 3 months with no interference, while a stage II surgery was needed.

**Diagnosis:**

Probing depth measurements suggested that the mesial, medial, and distal widths of buccal keratinized mucosa within the edentulous area were 0.5, 0.5, and 1 mm, respectively, which were insufficient to maintain the health of peri-implant tissues.

**Interventions:**

Keratinized mucosa augmentation guided by XCM membranes was performed to increase the inadequate buccal keratinized mucosa.

**Outcomes:**

After 2 months of healing, the widths of mesial, medial, and distal buccal keratinized mucosa were 4, 3, and 3 mm, respectively, and the thickness of the augmented mucosa was 4 mm. Then a stage II surgery was followed. The patient was satisfied with the outcomes of keratinized mucosa augmentation.

**Lessons:**

Keratinized mucosa augmentation guided by double XCM membrane technique can be applied to cases with keratinized mucosa width within 2 mm around implants.

## 1 Introduction

The contour reduction of bone is inevitable after tooth extraction, as a reaction to surgical trauma and the absence of vascular supply as well functional stimulation ^[1]^. Meanwhile, soft tissues attached to bone increasingly atrophies, which leads to insufficient keratinized mucosa and shallow vestibular sulcus.^[1,2]^ These changes will further increase the difficulty of wound closure during bone augmentation, and increases the risk to the long-term peri-implant health.^[3]^

At present, it is believed that the width of keratinized mucosa ≥2 mm is essential to maintain peri-implant gingival health.^[4]^ Various studies suggested that a reduced width of keratinized mucosa is a potential risk factor for peri-implant diseases.^[5,6]^ Nowadays, harvesting of free gingival graft (FGG) from the palate is a common and effective technique for soft tissue augmentation, whereas, many patients are reluctant to accept this surgery due to an additional surgical area.^[7,8]^ Problems following FGG procedure include pain, paresthesia, oral ulcer, injury to nerves and vessels, infection and excessive bleeding.^[9–11]^ Xenogeneic collagen matrix (XCM) membrane is used as a barrier for tissue regeneration, which is derived from natural animal skin tissue that completely removes the epidermal layer and cellular components by physical and chemical methods. XCM is a bilayer consisting of an outer compacted layer and inner porous matrix layer. It does not damage the original natural 3D scaffold structure and retains the natural extracellular matrix (ECM) containing the collagen network in dermis.^[12]^ Moreover, XCM is applied to repair gingival papilla in periodontal surgery,^[13]^ and as a scaffold in dura repair, which can induce the healing of the dura, reduce the inflammatory reaction, and promote cell proliferation and vascular growth.^[14]^

To avoid additional surgery and overcome other shortcomings of FGG, clinicians have been devoted to propose alternative techniques and materials for augmenting periodontal or peri-implant keratinized mucosa, including enamel matrix derivative, acellular dermal matrix, barrier membranes, and collagen matrix.^[15]^ But there is no consensus till date. In this case, we described a modified and simple keratinized tissue augmentation technique to obtain adequate vertical and horizontal keratinized tissue in the posterior region of mandible. This process increased the width of keratinized mucosa and restored the shallowed vestibular groove caused by long-term teeth loss. As a result, the patient experienced less therapeutic trauma and obtained promising results. The mesial, medial, and distal vertical increments of keratinized mucosa were 4, 3, and 3 mm respectively. The success of this case study suggests that we can further explore the effectiveness of keratinized mucosa augmentation technique guided by double XCM membranes.

## 2 Case report

A 66-year-old healthy female patient with loss of left mandibular first molar and second molar (FDI #36, #37) for 5 years consulted the department of oral implantology. She claimed that she had neither systematic diseases nor history of bruxism. The horizontal and vertical prosthetic spaces of the edentulous area were 18 and 7 mm, respectively, which was sufficient for implant prosthetics with an anatomical design, Examination by cone-beam computer tomography (CBCT) showed that the available bone height of #36 and #37 were 11 and 12 mm, respectively, in addition, the width of their crest were 8 and 7 mm, respectively. It was sufficient for complete implantation. Unfortunately, the buccal keratinized mucosa widths of #36 and #37 were inadequate, which required further keratinized mucosa augmentation. Based on the patient's condition, we arranged a regular implant surgery at first and a keratinized mucosa augmentation before stage II surgery.

Before surgery, the patient was informed about the operative risk and complications, and written consent was obtained from the patient for publication of this case report and accompanying images. Mouth rinsing was performed 3 times with 0.2% chlorhexidine solution. Under local infiltration anesthesia with articaine, a linear incision was made on the alveolar ridge crest of #36 and #37. Implant socket preparation was drilled step by step under permanent cooling with 0.9% saline, and 2 Straumann SLActive bone level implants (Φ4.1 mm × 10 mm, Straumann, Switzerland) were placed with a final insertion torque of 35 N/cm.

**Figure 1:**
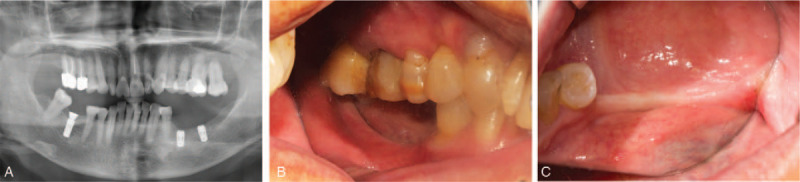
(A) 3 months after implant surgery, X-ray examination showed that good osseointegration was obtained. (B, C) Clinical oral observation showed that the keratinized mucosa is less than 2 mm.

Three months after the surgery, good osseointegration was observed by X-ray examination (Fig. 1A). Probe measurements showed that the mesial, medial and distal buccal keratinized mucosa were 0.5, 0.5, 1 mm, which were inadequate to maintain the health of peri-implant tissue (Fig. 1B and C).

**Figure 2:**
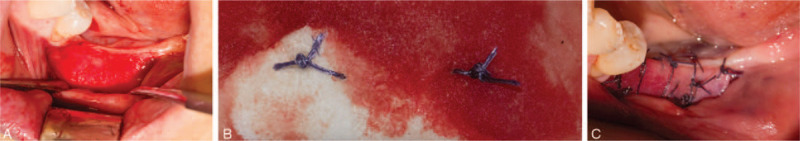
(A) A transverse incision was made along buccal mucogingival junction. Semi-gingival flap was stripped apically with elevator. (B) Double xenogeneic collagen matrix membranes were stitched together. (C) Double-absorbable membranes (2.5 cm × 2 cm, 0.69 mm thickness, Haiao, China) were fixed by sutures to cover the exposed periosteum.

In consideration of patient's reluctance for FGG, we devised to use double-absorbable XCM membranes for keratinized mucosa augmentation, which might obtain a thicker mucosa. Before the operation, the patient rinsed with 0.12% chlorhexidine mouthwash for 3 minute/time for 3 times. Under local infiltration anesthesia with articacine, a transverse incision was made along the buccal mucogingival junction. Semi-gingival flap was stripped apically with an elevator (Fig. 2A). Double-absorbable membranes (2.5 cm × 2 cm, 0.69 mm thickness, Haiao, China) were fixed by sutures to cover the exposed periosteum. In brief, 2 membranes were sutured layer by layer extra-orally, the porous matrix layer of the second membrane was brought into contact with the compacted layer of the first membrane. Then the edges of this double XCM membrane were inserted under the above mentioned semi-gingival flap, while the compacted layer of the second membrane was exposed to oral environment. Interrupted sutures were then applied to maintain stability (Fig. 2B and C).

**Figure 3:**
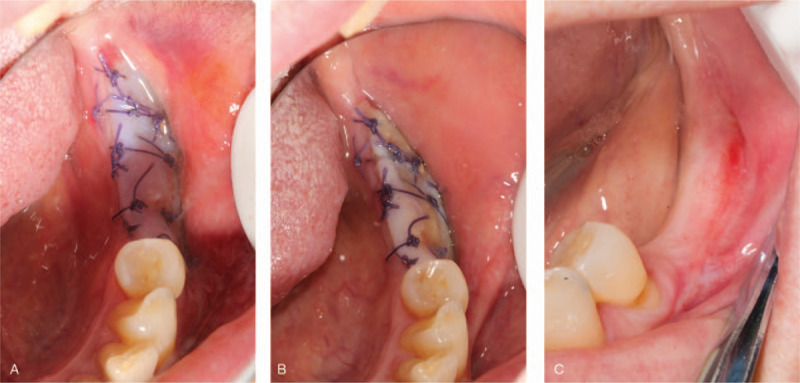
Healing situation (A) 2 days, (B) 4 days, (C) 1 month.

**Figure 4:**
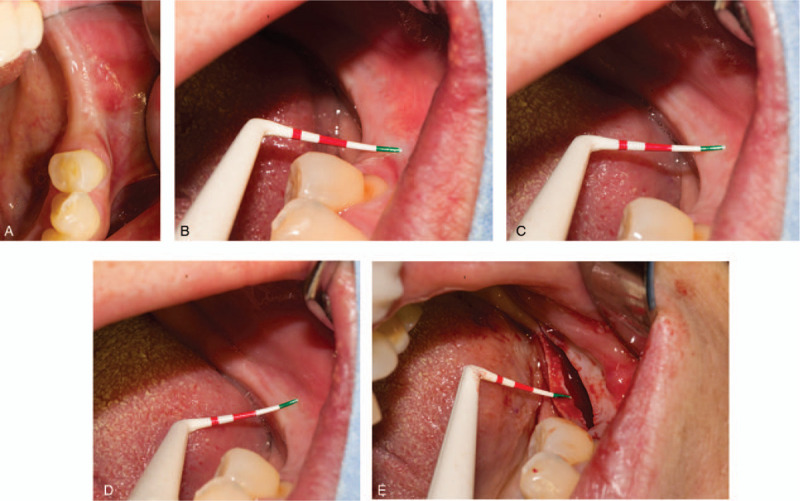
Stage II surgery (A) Keratiniazed mucosa is health and enough from occlusal view (B, C, D) Mesial, middle, distal width of buccal keratiniazed mucosa measured by probe was 4, 3, 3 mm. (E) The thickness of gingiva was 4 mm.

**Figure 5:**
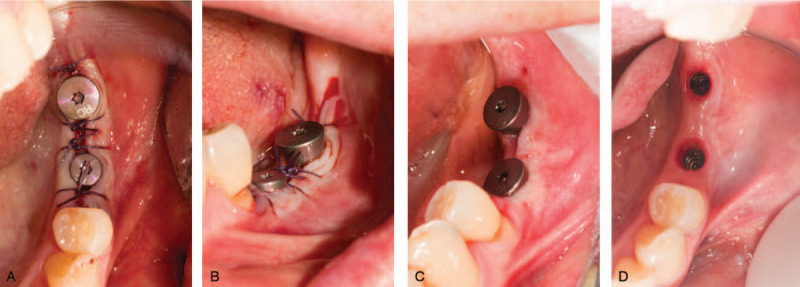
(A,B) Occlusal and buccal view of keratinized mucosa after stage II surgery (C) 7 days later (D) Gingival cuff wrapped around enough keratinized mucosa.

Regular follow-up visits were arranged at 2 days, 4 days and 1 month (Fig. 3). Stage II surgery was performed after 2 month wound healing (Fig. 4A). According to radiographic examinations and probe measurements, osseointegration was satisfactory and keratinized tissue volume was sufficient for next-stage prosthesis manufacture. The widths of mesial, medial, and distal buccal keratinized mucosa were 4, 3 and 3 mm, respectively (Fig. 4B, C, and D). The thickness of gingiva was 4 mm (Fig. 4E). Post-surgery suture and healing condition is showed in Fig. 5 A, B, and C. The patient was satisfied with the healing process as well as the final outcomes (Fig. 5D).

## 3 Discussion

Dental implant is a novel prosthetic technique aimed to simulate the physiological and anatomical structure of nature teeth. Compared with the natural periodontal tissue, the soft tissue around the implant contains fewer cells and blood vessels, and this feature is histologically similar to scar tissues with abundant collagen and few cell matrix. The implant is fixed in the alveolar bone with no cementum nor periodontal ligament around it. The gingival perforation part above the alveolar bone is surrounded by connective tissue bands. The fibers are mostly circular. The soft tissue around the implant is composed of epithelium, which adheres to the abutment or the implant shoulder. Natural tooth possesses functional connective tissue attachment of Sharpey fibers embedded in its unique cementum, whereas the surface of the implant has no such feature, which makes the soft tissue around the implant less compact and tensile than natural tooth. Owing to keratinized tissue's compactness and stability, keratinized mucosa around the implant is particularly important.^[16]^ Nowadays in implant dentistry, besides the pursuit of stable osseointergration, acquiring sufficient keratinized tissues is equally important to maintain peri-implant aesthetic and long-term health for both patients and clinicians.^[17,18]^

In a 10-year results of a prospective comparative study, Roccuzzo and Grasso^[19]^ found that implants which were not surrounded by keratinized mucosa were more prone to plaque accumulation and soft-tissue recession, even in those patients who exercised sufficient oral hygiene and received adequate supporting periodontal therapy. FGG is generally applied in the implant site to improve the deficiency of keratinized mucosa.^[20]^ Basegmez et al ^[21]^ indicated that the application of FGG is an effective method for increasing the width of attached mucosa (average 2.36 mm of 32 implants). In addition, Buyukozdemir and Berker ^[22]^ reported that the FGG group was better than the control group (without graft) in terms of gingival index at 1, 3, 6 months. Moreover, the use of FGG decreased the peri-implant sulcular fluid (PISF) volume, PISF IL-Iβ concentration, and bone loss. However, the complications, such as bleeding, and infections would occur due to free gingiva obtained from the maxilla palate. Patients tend to be reluctant and choose alternative methods because of fear of pain and the above-mentioned complications. In addition, the augmented soft tissue differs from the surrounding original gingival tissue in terms of color and texture,^[23]^ which limits its application in the aesthetic area.

To reduce the risks and disadvantages of FGG, clinicians have tried to use XCM for alternative free gingival graft. XCM provides cells and fibers with three-dimensional stent and growth space to adhere and proliferate to form sufficient keratinized mucosa. Sanz and Lorenzo ^[24]^ observed that the free connective tissue graft (CTG) attained a mean width of keratinized tissue of 2.6 mm, while for the collagen matrix (CM) it was 2.5 mm; but these differences were insignificant. Meanwhile, the characteristic of newly formed keratinized tissue is in accordance with the surrounding original tissue. The mechanism for this may be the kinds of cells and cytokines that induce new tissues, gradually form from the margin of the original tissue.

In the present case, after implant placement, stable osseointergration was obtained, but keratinized mucosa was deficient. Considering that soft tissue augmentation before prosthesis will obtain better outcome than after prosthesis placement, and that the patient is too sensitive to accept FGG therapy, we decided to use collagen matrix membrane graft plus apically positioned flap (APF) therapy. Basing on conventional technique, we modified single membrane to double membrane to strengthen the barrier function, and prolong the time of membrane degradation and cell proliferation. Moreover, double membranes would induce thicker tissue formation. The keratinized mucosa volume increased by at least 3 mm, meanwhile, APF caused deeper vestibular sulcus, and loosened incongruously pulled muscle. The patient also reported less discomfort on chewing and brushing, possibly due to the increased food overflow area and harmonious muscle movement.

In conclusion, the double-absorbable XCM membranes technology can be applied to soft tissue augmentation in cases with keratinized mucosa width less than 2 mm. This technique has several advantages, including fewer operative site, simplified surgical procedures, decreased risks of pain and infection, improved oral functional comforts, and promising aesthetic outcomes.

## Author contributions

**Conceptualization:** Chunyu Han, Weiyan Meng.

**Data curation:** Yuyang Li.

**Funding acquisition:** Weiyan Meng.

**Methodology:** Qing Cai.

**Resources:** Baosheng Li, Weiyan Meng.

**Software:** Haina Yu, Mengxiao He.

**Writing – original draft:** Chunyu Han, Weiyan Meng.

**Writing – review & editing:** Chunyu Han, Baosheng Li, Yanqun Liu, Weiyan Meng.
